# Supplementary material for: Risk Stratification and Clinical Characteristics of Patients with Late Recurrence of Melanoma (>10 Years)
Source: J Clin Med. 2022 Apr 5;11(7):2026. doi: 10.3390/jcm11072026 (PMC9000041; doi:10.3390/jcm11072026)
Supplement: Supplementary file 1 [file jcm-11-02026-s001.zip › jcm-1651048-supplementary.pdf]

Supplementary Materials

Figure S1. Immunohistochemical staining pigmented melanoma**.**

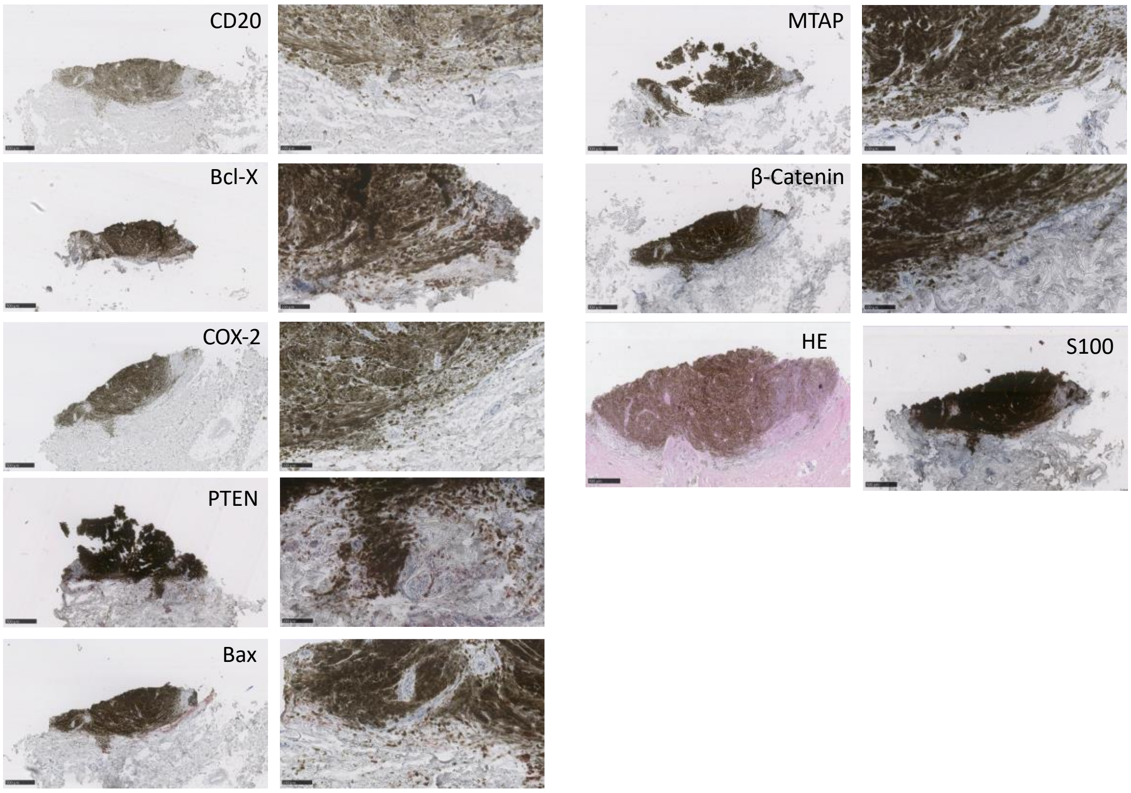

Table S1. Differential blood count of entire study collective, divided into normal, increased or decreased.

**Blood count at time of metastatic disease**		**Total**		**Male**		**Femal**	
***n* (%)**		**18 (100%)**		**9 (50%)**		**9 (50%)**	
		absolut	prozentual	absolut	prozentual	absolut	prozentual
**Leucocytes [exp9/L]**	Mean	8.3	-	9.3	-	7.4	-
	Median	7.8	-	8.3	-	7.6	-
n (%)	Decreased	0	-	0	-	0	-
	Normal	14 (77.8%)	-	6 (66.7%)	-	8 (88.9%)	-
	Increased	4 (22.2%)	-	3 (33.3%)	-	1 (11.1%)	-
**Lymphocytes [exp9/L]**	Mean	1.4	18.1%	1.6	19.60%	1.1	16.60%
	Median	1.3	16.90%	1.3	18.70%	1.2	16.20%
n (%)	Decreased	4 (22.2%)	10 (55.6%)	0	4 (44.4%)	4 (44.4%)	6 (66.7%)
	Normal	14 (77.8%)	8 (44.4%)	9 (100%)	5 (55.6%)	5 (55.6%)	3 (33.3%)
	Increased	0	0	0	0	0	0
**Eosinophil granulocytes [exp9/L]**	Mean	0.1	1.70%	0.2	2.00%	0.1	1.40%
	Median	0.1	1.20%	0.1	1.20%	0.1	1.10%
n (%)	Decreased	-	-	-	-	-	-
	Normal	18 (100%)	17 (94.4%)	9 (100%)	8 (88.9%)	9 (100%)	9 (100%)
	Increased	0	1 (5.6%)	0	1 (11.1%)	0	0
**Neutrophil granulocytes [exp9/L]**	Mean	6.1	71.30%	6.7	68.80%	5.6	73.60%
	Median	5.7	72.30%	6	68.70%	5.6	74.70%
n (%)	Decreased	0	0	0	0	0	0
	Normal	12 (66.7%)	13 (72.2%)	5 (55.6%)	8 (88.9%)	7 (77.8%)	5 (55.6%)
	Increased	6 (33.3%)	5 (27.8%)	4 (44.4%)	1 (11.1%)	2 (22.2%)	4 (44.4%)
**Thrombocytes [exp9/L]**	Mean	274.9	-	286	-	263.8	-
	Median	280.5	-	281	-	276	-
n (%)	Decreased	1 (5.6%)	-	0	-	1 (11.1%)	-
	Normal	15 (83.3%)	-	8 (88.9%)	-	7 (77.8%)	-
	Increased	2 (11.1%)	-	1 (11.1%)	-	1 (11.1%)	-

Table S2. Comparison ultra-late recurrence of melanoma (>15 years between primary melanoma and metastasis) and late recurrence melanoma (10-15 years between primary melanoma and metastasis).

	**Late Recurrence Melanoma (10–15 years)**	**Ultra-Late Reccurence Melanoma (>15 years)**
n	24	12
Male *n* (%)	10 (41.7%)	8 (66.7%)
Age at diagnosis [years] (Median)	51.9 (50.6)	50.3 (50.7)
Age at metastasis [years] (Median)	63.6 (62.8)	71.5 (72.6)
Time period until metastatic disease [years] (Median)	11.7 (11.7)	21.2 (19.0)
Breslow's depth [mm] (Median)	1.7 (1.5)	1.9 (1.8)
Localization of primary melanoma:		
Head/Neck *n* (%)	3 (12.5%)	2 (16.7%)
Trunk *n* (%)	16 (66.7%)	3 (25%)
Upper extremitiy *n* (%)	3 (12.5%)	2 (16.7%)
Lower extremitiy *n* (%)	2 (8.3%)	5 (41.7%)
S100 at time of metastatic disease, [ng/mL] (Median)	0.76 (0.07)	0,42 (0.11)
LDH at time of metastatic disease, [µkat/L] (Median)	5.8 (4.3)	5.6 (5.9)
